# Buprenorphine/naloxone – one formulation that doesn’t fit all: a case report

**DOI:** 10.1186/s12954-024-01054-2

**Published:** 2024-07-30

**Authors:** Hannan M. Braun, Jessica L. Taylor, Sarah Axelrath

**Affiliations:** 1 Division of General Internal Medicine, Denver Health and Hospital Authority, 301 W 6th Ave, Denver, 80204 CO USA; 2 Department of Internal Medicine, University of Colorado School of Medicine, Aurora, CO USA; 3 General Internal Medicine, Boston University Chobanian & Avedisian School of Medicine, Boston, USA; 4 Grayken Center for Addiction, Boston Medical Center, Boston, USA; 5 Colorado Coalition for the Homeless, Denver, CO USA

**Keywords:** Buprenorphine, Naloxone, Opioid use disorder, Stigma, Mistrust

## Abstract

**Background:**

Sublingual buprenorphine, approved for treatment of opioid use disorder since 2002, is most commonly available in co-formulation with naloxone. Naloxone is an opioid antagonist minimally absorbed when sublingual (SL) buprenorphine/naloxone is taken as prescribed; it is thought to reduce potential for misuse via intravenous administration. However, growing data and clinical experience demonstrate that previously accepted assumptions about the pharmacokinetics of these medications may not apply to all patients.

**Case presentation:**

We present a patient whose adverse post-administration side effects on SL buprenorphine/naloxone resolved with transition to SL buprenorphine monoproduct.

**Discussion:**

Naloxone can be detected in nearly all patients taking SL buprenorphine/naloxone, though with apparent variability in clinical effect. In a minority of patients, naloxone can contribute to adverse and potentially treatment-limiting side effects. Furthermore, the naloxone component is commonly misunderstood by patients and providers and can foster mistrust in the therapeutic relationship if providers are perceived to be withholding a more tolerable formulation. Prescribers should have a low threshold to offer buprenorphine alone when clinically appropriate.

## Background

Buprenorphine is a gold standard treatment for patients with opioid use disorder (OUD), a treatable chronic medical disorder. Buprenorphine is a partial µ-opioid receptor agonist with a long half-life and is associated with a reduced opioid overdose fatality risk as well as decreased rates of overall mortality and hepatitis C and HIV risk behaviors [1, 2].

When approved to treat OUD in 2002, sublingual (SL) buprenorphine was co-formulated with naloxone, an opioid antagonist, with the aim of reducing the potential for misuse. This strategy was employed in the 1980s with the coformulation of pentazocine and naloxone to deter injection of pentazocine [3]. The dual product was based on the premise that buprenorphine has comparatively high bioavailability with sublingual administration (35–55%), as compared to naloxone (< 10%) [4]. Therefore, it was assumed that naloxone would be inert if taken sublingually and not interfere with the action of buprenorphine. However, if injected, naloxone in a 4:1 buprenorphine: naloxone ratio [5] would become pharmacologically active and block the agonist effect of buprenorphine—preventing euphoria and/or precipitating withdrawal, thus discouraging patients from parenteral administration. This reasoning for inclusion of co-formulated buprenorphine/naloxone as an ‘anti-abuse’ mechanism is taught to health professions students, providers, and patients [6]. Buprenorphine/naloxone is commonly the preferred formulation of insurance plans; among US commercial formularies in 2021, 93.1% covered generic buprenorphine/naloxone films without prior authorization, compared with 75.8% for generic buprenorphine tablets [7].

Despite low bioavailability with SL administration, naloxone is detectable in nearly all patients taking sublingual buprenorphine/naloxone, [8, 9] though the value of urine naloxone that is clinically meaningful remains unknown. Even with detectable naloxone levels, the combined buprenorphine/naloxone is still effective treatment for OUD because of the relatively higher binding affinity of buprenorphine at the µ-opioid receptor as compared to naloxone.

While many patients tolerate buprenorphine/naloxone well in spite of low but detectable naloxone levels, a growing body of pre-clinical data and clinical experience [10–12] demonstrate that some patients do experience nausea, headache, or anxiety in the 30–60 min after taking the medication—symptoms that often resolve with a transition to the monoproduct. We present a 44-year-old woman who experienced treatment-limiting nausea from the naloxone component of buprenorphine/naloxone. The patient signed written informed consent for publication.

## Case presentation

A 44-year-old woman with a history of severe OUD in remission, moderate alcohol use disorder in remission, and tobacco use disorder presented to an outpatient addiction medicine practice for follow-up.

She initiated opioid use in her 30s with non-prescribed oral oxycodone and quickly met criteria for a severe use disorder. She initiated SL buprenorphine/naloxone at age 40 and entered OUD remission on buprenorphine/naloxone 4-1 mg twice per day; however, she experienced nausea and anxiety for approximately 30–45 min after each SL administration, which did not abate with continued use. Despite this side effect, she remained in treatment.

In clinic, she reported no change in her post-administration nausea. She reported no use of other opioids or non-prescribed substances. Review of her urine toxicology show buprenorphine and its metabolite norbuprenorphine at expected ratios and naloxone at concentrations of 21–849 ng/mL, consistent with buprenorphine/naloxone adherence [8]. Urine toxicology tests were negative for other opioids. Due to her nausea, she was transitioned to SL buprenorphine monoproduct, 4 mg twice daily, and her symptoms resolved. She remains in treatment.

## Discussion

### Complex pharmacology drives clinical challenges

The pharmacology of SL buprenorphine/naloxone is complex, contributing to diverse misunderstandings about its initiation and potential side effects. Because naloxone is also used – via different routes and doses - to reverse opioid overdose, people may incorrectly assume that the naloxone component is highly bioavailable and responsible for precipitating opioid withdrawal and “blocking” the euphoric effects of other opioids. This can make patients reluctant to consider a potentially effective treatment for them and can increase their risk of precipitated opioid withdrawal if they attempt to initiate buprenorphine prematurely using the mono-product.

On the other hand, providers often taught an oversimplified understanding of SL naloxone as minimally bioavailable, serving solely an “anti-abuse” function with no expected side effects in patients who take SL buprenorphine/naloxone as prescribed. Prescribers who are not aware of the potential inter-person variability in SL naloxone bioavailability and the potential for side effects in some patients may therefore doubt the veracity of reported side effects or the relationship of symptoms to the SL buprenorphine/naloxone. Patients who request a SL buprenorphine mono-product may also be suspected of an intent to use their medication via non-prescribed routes (e.g., injection) or to divert it.

### What are potential harms of the SL buprenorphine mono-product?

If patients are transitioned to SL buprenorphine without SL naloxone, might they be more prone to inject it? Available literature cannot definitively answer this question, though the introduction of SL buprenorphine/naloxone to replace SL buprenorphine in the Malaysian market did not decrease buprenorphine injection [13]. Furthermore, the combination product does not eliminate use by non-prescribed routes, as intravenous use of SL buprenorphine/naloxone is well described [13, 14]. Some patients report they are able to inject SL buprenorphine/naloxone without precipitated withdrawal, presumably due to buprenorphine’s relatively higher affinity for the µ-opioid receptor, the relatively lower dose of naloxone, and naloxone’s shorter half-life. While studies have shown that the addition of IV naloxone to IV buprenorphine attenuates positive subjective effects, [15–18] this reduction was often temporary [16, 18, 19]. While we are not aware of data on the relative risks of injecting buprenorphine compared to illicitly-manufactured fentanyl analogs, in the context of the current poisoned drug supply in the United States, patients who choose to inject buprenorphine, a partial opioid agonist with a long-half life, may be accurately assessing their overdose risk to be lower than it would be with injection of fentanyl.

The SL buprenorphine mono-product does carry a higher street value than SL buprenorphine/naloxone, [20] and diversion and prescription opioid misuse can be sources of harm. For example, in Finland where the illicit drug market consists almost exclusively of imported buprenorphine products, over a quarter of buprenorphine-positive fatal overdoses involved IV administration—almost entirely (98.7%) in combination with other central nervous system depressants such as benzodiazepine and alcohol [21]. Conversely, in the US where the overdose crisis is driven by illicitly manufactured fentanyl analogs, diverted buprenorphine is often used for self-treatment of OUD where access to buprenorphine treatment is inadequate, [22] and diverted buprenorphine reduces risk of overdose [23].

It is the responsibility of all controlled substance prescribers to take steps to minimize medication use via non-prescribed routes and diversion. However, we believe that our clinical tools—evaluating patient-reported symptoms and physical exam signs, setting clear expectations, and utilizing prescription monitoring programs, urine drug screens with buprenorphine metabolites when indicated, pill counts, or shortened prescriptions, and considering injectable buprenorphine formulations—can be employed in a thoughtful, stepwise approach when patients may be struggling or demonstrate behaviors concerning for buprenorphine injection or diversion. Continuing to prescribe SL buprenorphine/naloxone in spite of patient-reported adverse can also reflect a stigmatizing belief that people with OUD are likely to inject or divert their medication without the naloxone guardrail, which can harm the therapeutic alliance between patient and provider. The potential for diversion, which exists for all controlled substances, is not a reason to limit application of an effective medication in individual situations, such as the case presented above, where the benefits outweigh the risks.

### What are the potential harms of underutilizing SL buprenorphine?

Until her switch to the SL buprenorphine mono-product, our patient was able to sustain OUD remission on SL buprenorphine/naloxone in spite of naloxone-associated side effects. However, in other cases, naloxone-associated side effects may contribute to premature treatment discontinuation and/or a return to non-prescribed opioid use. Provider reluctance to offer the SL buprenorphine mono-product - possibly rooted in misunderstanding of pharmacology, outsized fears of the harms of the mono-product, stigma, or concerns about coverage barriers - can foster unnecessary mistrust between patient and provider, damaging the therapeutic alliance. Given the urgency of the opioid overdose crisis, these are barriers we should not tolerate.

### Buprenorphine mono-product availability

Buprenorphine for OUD treatment, without co-formulated naloxone, is currently available as a weekly or monthly subcutaneous injection and a SL tablet. A SL buprenorphine film does not currently exist on the market. Some patients prefer a film formulation over a tablet for faster dissolving, and newer low-dose buprenorphine induction protocols require cutting a film into small pieces, which is difficult or impossible with a tablet. We would be pleased to see a SL buprenorphine film introduced as an option for treating OUD, which could facilitate treatment for a subset of patients.

Many of our patients who have experienced side effects related and unrelated to the naloxone component of SL buprenorphine/naloxone have successfully transitioned to injectable buprenorphine. However, some patients do not desire injections or rely on the routine of daily medication to maintain OUD remission; additional implementation and access barriers remain [24] for subcutaneous buprenorphine related to transportation, insurance coverage, and availability of providers.

## Conclusion

Buprenorphine is associated with profound reductions in mortality among patients with OUD [1] and it is among the most effective medications we can prescribe in primary care, with a number needed to treat to prevent a death in the year following overdose of approximately 52 [25]. While the majority of patients tolerate SL buprenorphine/naloxone well, the naloxone component adds minimal clinical benefit for the overwhelming majority of patients who take SL buprenorphine/naloxone as prescribed, and it risks limiting treatment in the subset of patients who experience naloxone-related side effects.

Considering demonstrated inter-patient variability in SL naloxone bioavailability, increasing experience with patients like ours who report resolution of side effects with a transition from SL buprenorphine/naloxone to the buprenorphine mono-product, and the potential harms of withholding effective OUD treatment amidst the highest rates of opioid overdose death in US history, we recommend providers consider a transition to buprenorphine alone if naloxone-related side effects are suspected. We encourage insurances to cover buprenorphine mono-products without prior authorizations or other onerous administrative barriers.

## Data Availability

No datasets were generated or analysed during the current study.

## Abbreviations

OUD: opioid use disorder
SL: sublingual

## References

[1] Santo T Jr, Clark B, Hickman M, et al. Association of Opioid Agonist Treatment with all-cause mortality and specific causes of death among people with opioid dependence: a systematic review and Meta-analysis. JAMA Psychiatry. 2021;78(9):979–93. 10.1001/jamapsychiatry.2021.0976. 34076676 10.1001/jamapsychiatry.2021.0976 PMC8173472

[2] MacArthur GJ, Minozzi S, Martin N, et al. Opiate substitution treatment and HIV transmission in people who inject drugs: systematic review and meta-analysis. BMJ. 2012;345:e5945. 10.1136/bmj.e5945. 23038795 10.1136/bmj.e5945 PMC3489107

[3] Baum C, Hsu JP, Nelson RC. The impact of the addition of naloxone on the use and abuse of pentazocine. Public Health Rep. 1987;102(4):426–9. 3112855 PMC1477863

[4] Harris DS, Mendelson JE, Lin ET, Upton RA, Jones RT. Pharmacokinetics and subjective effects of sublingual buprenorphine, alone or in combination with naloxone: lack of dose proportionality. Clin Pharmacokinet. 2004;43(5):329–41. 15080765 10.2165/00003088-200443050-00005

[5] Mendelson J, Jones RT. Clinical and pharmacological evaluation of buprenorphine and naloxone combinations: why the 4:1 ratio for treatment? Drug and Alcohol Dependence. 2003;70(2, Supplement):S29-S37. 10.1016/S0376-8716(03)00057-7. 10.1016/s0376-8716(03)00057-7 12738348

[6] PCSS. 8 Hour MOUD Training: Medications for Opioid Use Disorder. Presented at: Accessed December 9. 2023. https://education.sudtraining.org/Listing/8-Hour-Online-MAT-Waiver-Training-4607.

[7] Nguyen TD, Chua KP, Andraka-Christou B, Bradford WD, Simon K. Trends in Buprenorphine Coverage and prior authorization requirements in US Commercial formularies, 2017–2021. JAMA Health Forum. 2022;3(7):e221821. 10.1001/jamahealthforum.2022.1821. 35977219 10.1001/jamahealthforum.2022.1821 PMC9270692

[8] Heikman P, Häkkinen M, Gergov M, Ojanperä I. Urine naloxone concentration at different phases of buprenorphine maintenance treatment. Drug Test Anal. 2014;6(3):220–5. 10.1002/dta.1464. 23512803 10.1002/dta.1464

[9] Warrington JS, Warrington GS, Francis-Fath S, Brooklyn J. Urinary buprenorphine, Norbuprenorphine and naloxone concentrations and ratios: review and potential clinical implications. J Addict Med. 2020;14(6):e344. 10.1097/ADM.0000000000000676. 32530884 10.1097/ADM.0000000000000676

[10] Gregg J, Hartley J, Lawrence D, Risser A, Blazes C. The Naloxone Component of Buprenorphine/Naloxone: discouraging misuse, but at what cost? J Addict Med. 2023;17(1):7–9. 10.1097/ADM.0000000000001030. 35913990 10.1097/ADM.0000000000001030

[11] Blazes CK, Morrow JD. Reconsidering the usefulness of adding Naloxone to Buprenorphine. Front Psychiatry. 2020;11:549272. 10.3389/fpsyt.2020.549272. 33061915 10.3389/fpsyt.2020.549272 PMC7517938

[12] Grande LA. Prescribing the Buprenorphine Monoproduct for adverse effects of Buprenorphine-Naloxone. J Addict Med. 2022;16(1):4–6. 10.1097/ADM.0000000000000837. 33758111 10.1097/ADM.0000000000000837

[13] Bruce RD, Govindasamy S, Sylla L, Kamarulzaman A, Altice FL. Lack of reduction in Buprenorphine Injection after introduction of Co-formulated Buprenorphine/Naloxone to the Malaysian market. Am J Drug Alcohol Abus. 2009;35(2):68–72. 10.1080/00952990802585406. 10.1080/00952990802585406 PMC2791788 19212931

[14] Lofwall MR, Walsh SL. A review of buprenorphine diversion and misuse: the current evidence base and experiences from around the world. J Addict Med. 2014;8(5):315–26. 10.1097/ADM.0000000000000045. 25221984 10.1097/ADM.0000000000000045 PMC4177012

[15] Jones JD, Manubay JM, Mogali S, et al. Abuse liability of intravenous buprenorphine vs. buprenorphine/naloxone: importance of absolute naloxone amount. Drug Alcohol Depend. 2017;179:362–9. 10.1016/j.drugalcdep.2017.06.033. 28844013 10.1016/j.drugalcdep.2017.06.033 PMC10071507

[16] Comer SD, Sullivan MA, Vosburg SK, et al. Abuse liability of intravenous buprenorphine/naloxone and buprenorphine alone in buprenorphine-maintained intravenous heroin abusers. Addiction. 2010;105(4):709–18. 10.1111/j.1360-0443.2009.02843.x. 20403021 10.1111/j.1360-0443.2009.02843.x PMC3489277

[17] Weinhold LL, Preston KL, Farre M, Liebson IA, Bigelow GE. Buprenorphine alone and in combination with naloxone in non-dependent humans. Drug Alcohol Depend. 1992;30(3):263–74. 10.1016/0376-8716(92)90061-g. 1396108 10.1016/0376-8716(92)90061-g

[18] Comer SD, Collins ED. Self-administration of intravenous buprenorphine and the buprenorphine/naloxone combination by recently detoxified heroin abusers. J Pharmacol Exp Ther. 2002;303(2):695–703. 10.1124/jpet.102.038141. 12388653 10.1124/jpet.102.038141 PMC4079473

[19] Duke AN, Correia CJ, Walsh SL, Bigelow GE, Strain EC. Acute effects of intramuscular and sublingual buprenorphine and buprenorphine/naloxone in non-dependent opioid abusers. Psychopharmacology. 2010;211(3):303–12. 10.1007/s00213-010-1898-4. 20577717 10.1007/s00213-010-1898-4 PMC2941639

[20] Alho H, Sinclair D, Vuori E, Holopainen A. Abuse liability of buprenorphine-naloxone tablets in untreated IV drug users. Drug Alcohol Depend. 2007;88(1):75–8. 10.1016/j.drugalcdep.2006.09.012. 17055191 10.1016/j.drugalcdep.2006.09.012

[21] Kriikku P, Häkkinen M, Ojanperä I. High buprenorphine-related mortality is persistent in Finland. Forensic Sci Int. 2018;291:76–82. 10.1016/j.forsciint.2018.08.010. 30170272 10.1016/j.forsciint.2018.08.010

[22] Cicero TJ, Ellis MS, Chilcoat HD. Understanding the use of diverted buprenorphine. Drug Alcohol Depend. 2018;193:117–23. 10.1016/j.drugalcdep.2018.09.007. 30359928 10.1016/j.drugalcdep.2018.09.007

[23] Carlson RG, Daniulaityte R, Silverstein SM, Nahhas RW, Martins SS. Unintentional drug overdose: is more frequent use of non-prescribed buprenorphine associated with lower risk of overdose? Int J Drug Policy. 2020;79:102722. 10.1016/j.drugpo.2020.102722. 32311513 10.1016/j.drugpo.2020.102722 PMC9387534

[24] Morgan JR, Assoumou SA. The limits of innovation: directly addressing known challenges is necessary to improve the real-world experience of novel medications for opioid use disorder. Acad Emerg Med. 2023;30(12):1285–7. 10.1111/acem.14814. 37793818 10.1111/acem.14814 PMC10841521

[25] Poorman E. The number needed to Prescribe — what would it take to Expand Access to Buprenorphine? N Engl J Med. 2021;384(19):1783–4. 10.1056/NEJMp2101298. 33983689 10.1056/NEJMp2101298

